# Genomic and Transcriptional Profiling of Chinese Melanoma Patients Enhanced Potentially Druggable Targets: A Multicenter Study

**DOI:** 10.3390/cancers15010283

**Published:** 2022-12-31

**Authors:** Yue Li, Baoming Wang, Chunyang Wang, Dandan Zhao, Zhengchuang Liu, Yanling Niu, Xiaojuan Wang, Wei Li, Jianhua Zhu, Houquan Tao, Tonghui Ma, Tao Li

**Affiliations:** 1 Harbin Medical University Cancer Hospital, Harbin 150040, China; 2 Jichenjunchuang Clinical Laboratory, Hangzhou 310022, China; 3 Key Laboratory of Gastroenterology of Zhejiang Province, Zhejiang Provincial People’s Hospital, People’s Hospital of Hangzhou Medical College, Hangzhou 310014, China; 4 Department of Surgery, Zhejiang Provincial People’s Hospital, People’s Hospital of Hangzhou Medical College, Hangzhou 310014, China; 5 Institute of Basic Medicine and Cancer (IBMC), Department of Bone and Soft-tissue Surgery, The Cancer Hospital of the University of Chinese Academy of Sciences, Chinese Academy of Sciences, Hangzhou 310022, China

**Keywords:** melanoma, DNA-NGS, RNA-NGS, sequence, target

## Abstract

**Simple Summary:**

Although multiple actionable genes have been identified in melanoma, 38–42% of patients are still not druggable based on current research. In our study, DNA-NGS and RNA-NGS were utilized to construct molecular profiles of a Chinese cohort of 469 melanoma patients. Up to 11.7% (7/60) of patients in the undruggable group could be recognized as actionable by DNA and RNA sequential sequencing. Additionally, the use of RNA-NGS enhanced the proportion of druggable fusions from 2.56% to 17.27%. In total, the use of RNA-NGS increased the druggable proportion from 75% to 78%. Our study systemically analyzed the genetic landscape of Asian melanoma and demonstrated how DNA and RNA sequential sequencing is essential in bringing clinical benefits to more melanoma patients.

**Abstract:**

Background: In contrast to Caucasian melanoma, which has been extensively studied, there are few studies on melanoma in Asian populations. Sporadic studies reported that only 40% of Asian melanoma patients could be druggable, which was much lower than that in Caucasians. More studies are required to refine this conclusion. Methods: Chinese melanoma patients (*n* = 469) were sequentially sequenced by DNA-NGS and RNA-NGS. The genomic alterations were determined, and potentially actionable targets were investigated. Results: Patients with potential druggable targets were identified in 75% of Chinese melanoma patients by DNA-NGS based on OncoKB, which was much higher than in a previous Asian study. *NRG1* fusions were first identified in melanoma. In addition, up to 11.7% (7/60) of patients in the undruggable group could be recognized as actionable by including RNA-NGS analysis. By comparing the fusion detection rate between DNA-NGS and RNA-NGS, all available samples after DNA-NGS detection were further verified by RNA-NGS. The use of RNA-NGS enhanced the proportion of druggable fusions from 2.56% to 17.27%. In total, the use of RNA-NGS increased the druggable proportion from 75% to 78%. Conclusions: In this study, we systemically analyzed the actionable landscape of melanoma in the largest Asian cohort. In addition, we first demonstrated how DNA and RNA sequential sequencing is essential in bringing clinical benefits to more patients with melanoma.

## 1. Background

The morbidity rate of melanoma has rapidly increased in recent years, causing more than 57,000 deaths worldwide in 2020 [1]. DNA-based next-generation sequencing (NGS) has been widely applied to decode the genetic landscape of melanoma [2,3,4,5]. With comprehensive molecular profiling, multiple actionable targets have been discovered; for example, *BRAF V600E* could be targeted with dabrafenib and trametinib, which have been approved by the FDA for pan-cancer treatment [6]. Furthermore, numerous oncogenic driver fusions could be inhibited with inhibitors in melanoma [7,8,9]. For example, tumors with *NRTK* fusions could be inhibited by TRK inhibitors [9]. Additionally, patients with a high tumor mutation burden (TMB-H) or high microsatellite instability (MSI-H) could be referred to immunotherapy [10,11,12,13].

Although multiple actionable genes have been identified in melanoma, 38–42% of patients are still not druggable based on current research [14,15]. In non-small-cell lung cancer (NSCLC), DNA and RNA sequential sequencing could bring clinical benefits to an additional 14% of patients in the driver-negative cohort [16]. This suggests that additional screening techniques need to be utilized. RNA-NGS is a great candidate to extend the druggable map. However, distinct from Western countries where RNA-NGS utility is now routine diagnosis, RNA sequencing is less common in China. Therefore, few studies have been performed on melanoma.

To identify underlying genomic targets with clinical significance, DNA-NGS and RNA-NGS were utilized to construct molecular profiles of a Chinese cohort of 469 melanoma patients. We systematically delineated the molecular genetic landscape of Chinese melanoma patients and stratified patients into druggable or undruggable groups based on actionable genes, TMB status, and MSI status. Furthermore, potential actionable genes were investigated in the undruggable group, and we demonstrated how RNA sequencing is essential in bringing benefits to more melanoma patients.

## 2. Methods

### 2.1. Patients Population

Tumor histological specimens (formalin-fixed paraffin-embedded tissues or frozen tissues) obtained from multi-centers (Harbin Medical University Cancer Hospital, Zhejiang Provincial People’s Hospital and Cancer Hospital of the University of Chinese Academy of Sciences (Zhejiang Cancer Hospital)) between March 2018 and May 2020 were used for NGS detection. This study has been approved by the Ethics Committee of the Zhejiang Cancer Hospital (IRB-2021-3). All patients were diagnosed via pathology, imaging, and clinical findings.

### 2.2. DNA Isolation and Targeted Sequencing

Genomic DNA was extracted from tumor samples using a QIAamp DNA FFPE Tissue Kit (Qiagen, Valencia, CA, USA) and fragmented to construct a library using KAPA Hyper Prep kits (KAPA, KK8504). DNA fragments of 200~400 bp were purified by an Agencourt AMPure XP Kit (Beckman Coulter, CA, USA). Fragmented genomic DNA libraries were constructed with the KAPA HTP Library Preparation Kit (Kapa Biosystems, Wilmington, MA, USA) according to the instruction manual. After determining the concentration by a Qubit dsDNA HS Assay kit, DNA libraries were analyzed using Onco PanScan™ (GenetronHealth; Beijing, China), which is an 825-gene panel that includes major tumor-related genes.

### 2.3. RNA Sequencing

Total RNA was isolated from FFPE samples using the AllPrep DNA/RNA Mini Kit (QIAGEN, Hilden, Germany). The RNA quality and quantity were determined by the Qubit RNA HS Assay (Thermo Fisher Scientific, Waltham, MA, USA) and the 4200 TapeStation System (Thermo Fisher Scientific, Waltham, MA, USA), respectively. Qualified RNA was used to synthesize double-stranded cDNA using a SuperScript VILO cDNA Synthesis kit (Thermo Fisher Scientific, Waltham, MA, USA) and an Abclonal Second Strand Synthesis Module (Abclonal, Wuhan, China) according to the recommended protocol. To prepare genomic libraries, the double-stranded cDNA was fragmented and constructed by a KAPA HTP Library Preparation Kit (KAPA Biosystems, Wilmington, MA, USA). The prepared libraries were captured with FusionCapture™ (Genetron Health, Beijing, China), which contains 395 cancer-related genes, and then subjected to Illumina NovaSeq6000 (Illumina, Inc., San Diego, CA, USA) for paired-end sequencing. The sequencing reads were mapped to UCSC hg19 through HISAT2-2.0.5, and FusionMap was used to identify gene fusions.

### 2.4. Statistical Analysis

All statistical analyses were conducted using GraphPad Prism (version 8.02) software. The results are expressed as the mean ± SEM. The data were analyzed using a t test or one-way ANOVA, followed by the appropriate post-hoc tests (GraphPad Prism 7, GraphPad, CA, USA).

## 3. Results

### 3.1. The Landscape of Somatically Actionable Genes Identified by DNA-NGS

**Figure 1:**
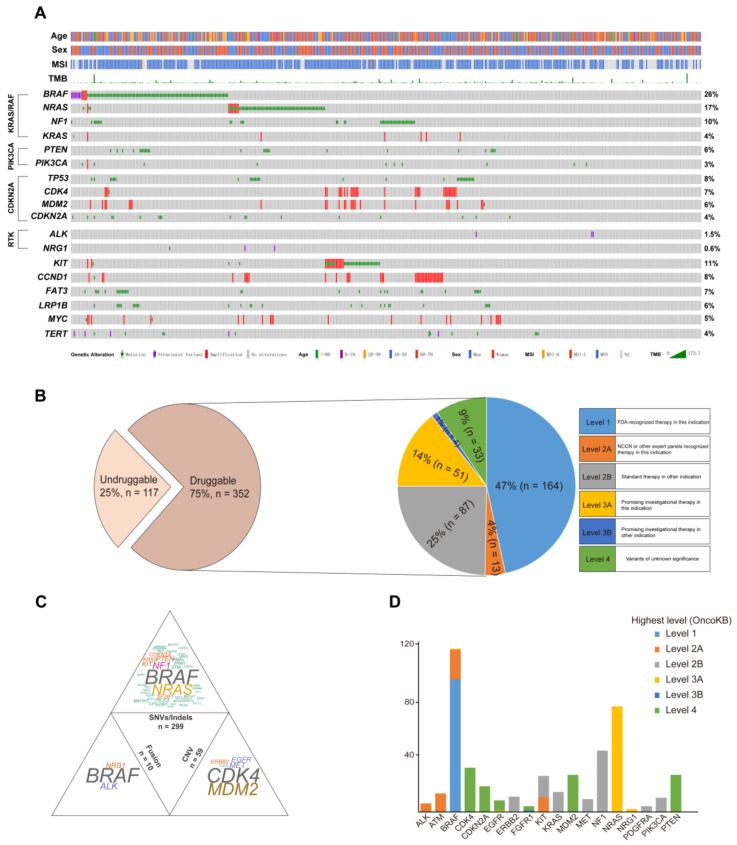
The characteristics of the actionable genes in melanoma patients identified by DNA sequencing. (**A**) The spectrum of the variations in the melanoma; (**B**) the percentage of patients that were actionable in the cohort; (**C**) the distribution of targetable mutations based on mutation types; and (**D**) the number of patients was plotted against the most actionable genes in the cohort.

Melanoma samples were obtained from 256 men (55%) and 213 women (45%) with ages ranging from 5 to 89 years (average age = 57 years). Collectively, 469 resected melanoma samples were screened by DNA sequencing. Numerous pathways were involved in the landscape. The *BRAF* mutation, *RAS* mutation (*NRAS, KRAS, HRAS*), and *NF1* mutation, which are included in the *KRAS/RAF* pathway, were found in 120 (26%), 115 (25%), and 46 (10%) samples, respectively. Mutations in *PTEN* and *PIK3CA* from the *PIK3CA* pathway were identified in 28 (6%) and 14 (3%) patients, respectively. The genes from the *CDKN2A* pathway were also analyzed, and *TP53* (8%, *n* = 36), *CDK4* (7%, *n* = 32), *MDM2* (6%, *n* = 27), and *CDKN2A* (4%, *n* = 20) were the most frequently mutated genes. Furthermore, two receptor tyrosine kinase fusion genes not related to the above pathways, *ALK* and *NRG1*, were found in our cohort, accounting for 1.5% (*n* = 7) and 0.6% (*n* = 3), respectively. In addition, other top six mutated genes in the cohort were *KIT* (11%, *n* = 50), *CCND1* (8%, *n* = 38), *FAT3* (7%, *n* = 32), *LRP1B* (6%, *n* = 26), *MYC* (5%), and *TERT* (4%) (Figure 1A). Apart from the mutated genes, the tumor mutation burden (TMB) was also calculated in all patients (mean TMB = 8.7), and 96 (20.5%, 96/469) participants presented high TMB (≥10). Microsatellite instability (MSI) status was identified in 374 patients, including 1 patient (0.3%, 1/374) with MSI-H.

To broadly and systematically evaluate the clinical utility of molecular profiling to guide therapy decisions in melanoma patients, OncoKB, a curated knowledge base of the oncogenic effects and treatment implications of somatic mutations that were clarified by the FDA, was used to group all mutations into levels of clinical actionability (http://oncokb.org/, accessed on 1 January 2022). Seventy-five percent (352/469) of patients had actionable genes after matching those genes with actionable mutations in OncoKB. The druggable patients were then grouped based on the highest level of drug response evidence in OncoKB. Approximately 47% (164/352) of patients could be classified as OncoKB level 1. This was followed by level 2 (29%, 100/352), level 3 (15%, 55/352), and level 4 (9%, 33/352) (Figure 1B).

To explore the specific variations involved in the druggable group, the potential actionable mutations were classified in a mutation-type-specific manner: SNV (single nucleotide variant)/Indel, CNV (copy number variant), and fusion, which were found in 85% (299/352), 17% (59/352), and 3% (10/352) of patients, respectively (Figure 1C). The patients with *BRAF*, *NRAS*, and *NF1* mutations were the top three in the SNV/Indel group, accounting for more than half of the total. In the CNV group, the proportions of *CDK4* and *MDM2* amplification were obviously predominant. Only three druggable tyrosine kinase fusion genes (*ALK*, *BRAF*, and *NRG1)* were identified at the DNA level, and the *BRAF* fusions were dominant (60%, *n* = 6) in the fusion group. Furthermore, the top genes in the three groups were analyzed, and the number of gene mutations was counted based on the highest actionable level from OncoKB. The top actionable mutation genes in the study were *BRAF*, *NRAS*, *NF1*, and *CDK4*. Most of the genes only had one highest level due to a single variation. However, *BRAF*, *FGFR1*, and *KIT* presented two highest levels because of various mutations (Figure 1D).

### 3.2. RNA-NGS Could Identify Actionable Fusions in Undruggable Melanoma Patients

**Figure 2:**
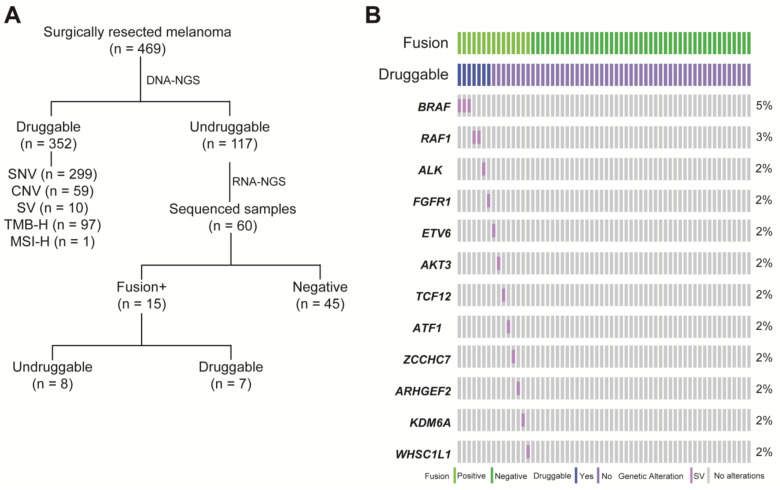

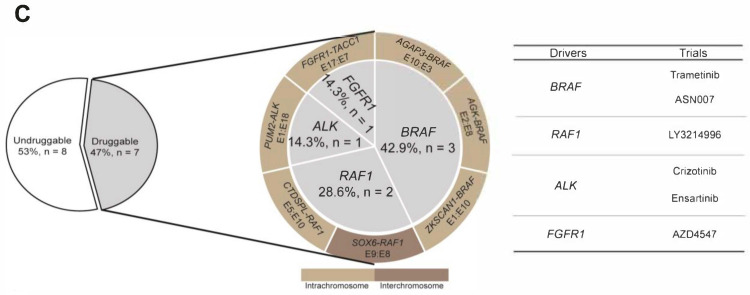
The fusion landscape of undruggable patients by RNA sequencing. (**A**) Description of the melanoma cohort by comprehensive DNA-seq and RNA-seq; (**B**) OncoPrint depicting fusions in the undruggable patients by Fusioncapture panel; and (**C**) pie chart summarizing the actionable fusions and matched therapy on RNA-level.

To explore additional opportunities for targeted therapy, the undruggable melanoma group identified by DNA-NGS was further sequenced by RNA-NGS. In total, 117 melanoma patients were identified as undruggable based on the DNA-NGS results. Sixty patients with enough samples were sequenced by RNA-NGS, and positive fusions were found in 15 samples (25%) (Figure 2A). These fusions involved multiple driver genes: *BRAF* (5%, *n* = 3), *RAF1* (3%, *n* = 2), *ALK* (2%, *n* = 1), *FGFR1* (2%, *n* = 1), *ETV6* (2%, *n* = 1), *AKT3* (2%, *n* = 1), *TCF12* (2%, *n* = 1), *ATF* (2%, *n* = 1), *ZCCHC7* (2%, *n* = 1), *ARHGEF2* (2%, *n* = 1), *KDM6A* (2%, *n* = 1), and *WHSC1L1* (2%, *n* = 1) (Figure 2B). Based on the RNA-NGS results, 47% (7/15) of the fusions could be druggable in the fusion-positive group, including *PUM2-ALK*, *FGFR1-TACC1, AGAP3-BRAF*, *AGK-BRAF*, *ZKSCAN1-BRAF*, *SOX6-RAF1*, and *CTDSPL-RAF1*. Most of the rearrangements occurred on the same chromosome, except for *SOX6-RAF1* (*chr11-chr3*). All fusions with druggable potential could be targeted by drugs from clinical trials (Figure 2C). In total, RNA-seq identified 11.7% (7/60) of patients in the undruggable group who carried potentially actionable targets.

### 3.3. RNA-NGS Could Significantly Enhance the Proportion of Fusions in Melanoma

**Figure 3:**
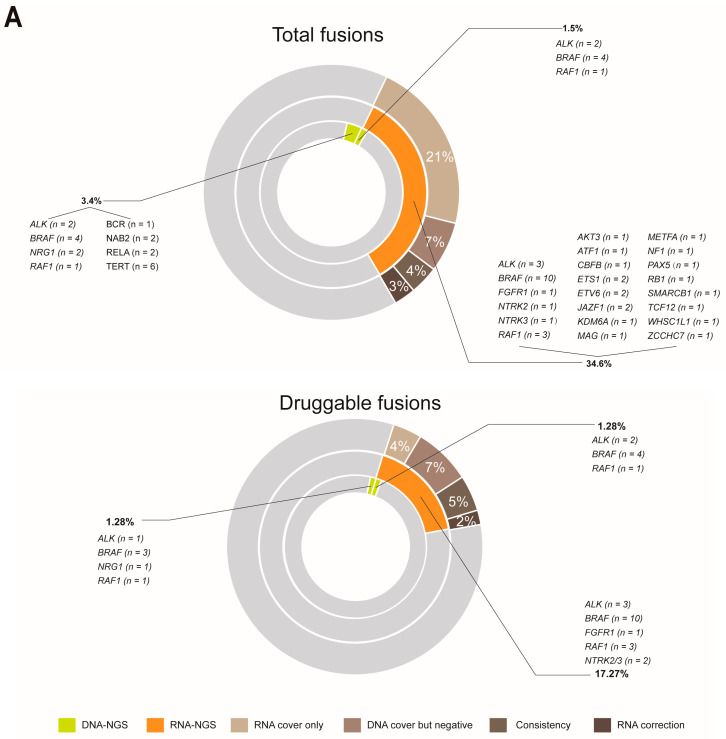

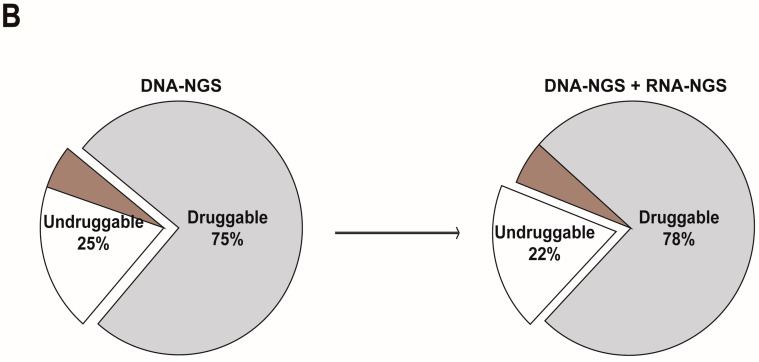
The comparison of fusion detection rates between DNA and RNA sequencing. (**A**) RNA sequencing increased the detection proportion of total and druggable fusions; and (**B**) RNA sequencing expanded the scope (enlarged the proportion) of druggable patients in melanoma.

At the DNA level, 27 fusions involving multiple fusion genes from the whole cohort were identified in 23 patients, which accounted for 4.9% of the cohort (23/469): *ALK* (*n* = 4), *BRAF* (*n* = 10), *NRG1* (*n* = 2), *RAF1* (*n* = 2), *BCR* (*n* = 1), *NAB2* (*n* = 2), *RELA* (*n* = 2), and *TERT* (*n* = 2) (Figure 3A). We explored how integrated DNA-NGS and RNA-NGS sequencing are essential in enhancing the proportion of druggable patients with melanoma through fusion detection. Patients with enough samples after DNA sequencing were detected by RNA-NGS (*n* = 110), regardless of whether they were druggable. At the RNA level, positive fusions were validated in 38 patients: *ALK* (*n* = 3), *BRAF* (*n* = 10), *FGFR1* (*n* = 1), *NTRK2* (*n* = 1), *NTRK3* (*n* = 1), *RAF1* (*n* = 3), *AKT3* (*n* = 1), *ATF1* (*n* = 1), *CBFB* (*n* = 1), *ETS1* (*n* = 2), *ETV6* (*n* = 2), *JAZF1* (*n* = 2), *KDM6A* (*n* = 1), *MAG* (*n* = 1), *METFA* (*n* = 1), *NF1* (*n* = 1), *PAX5* (*n* = 1), *RB1* (*n* = 1), *SMARCB1* (*n* = 1), *TCF12* (*n* = 1), *WHSC1L1* (*n* = 1), and *ZCCHC7* (*n* = 1) (Figure 3A). RNA-NGS enhanced the total fusion proportion from 4.9% (23/469) to 34.6% (38/110) (Figure 3A). If only the fusions with clinical potential were considered, the fusion proportion was 2.1% (10/469) at the DNA level; however, RNA-GS increased the percentage to 17.27% (19/110) (Figure 3A). Overall, RNA-NGS increased the percentage of druggable patients from 75% to 78% (Figure 3B).

The significant enhancement of fusion detection by RNA-NGS was attributed to the different designs of the DNA-NGS and RNA-NGS panels, which could be categorized into four groups: breakpoints that were covered by only the RNA-NGS panel (RNA cover only), breakpoints that were covered by DNA-NGS but failed to identify fusions (DNA cover but negative), the fusions that were verified by both DNA-NGS and RNA-NGS (Consistency), and the different partners that were validated by DNA-NGS and RNA-NGS (RNA correction) (Figure 3A). In the total fusion-positive cohort (34.6%), RNA cover accounted for only 21%, DNA cover but negative accounted for 7%, Consistency accounted for 4%, and the last 3% could be attributed to RNA correction (Figure 3A). Comparatively, in the druggable fusions group (17.27%), 4% was due to RNA cover only, 7% was due to DNA cover but negative, and Consistency and RNA correction accounted for 5% and 2%, respectively (Figure 3A).

### 3.4. Unreliability of the Fusion Breakpoint in Predicting Relevant Transcripts Based on DNA-NGS

**Figure 4:**
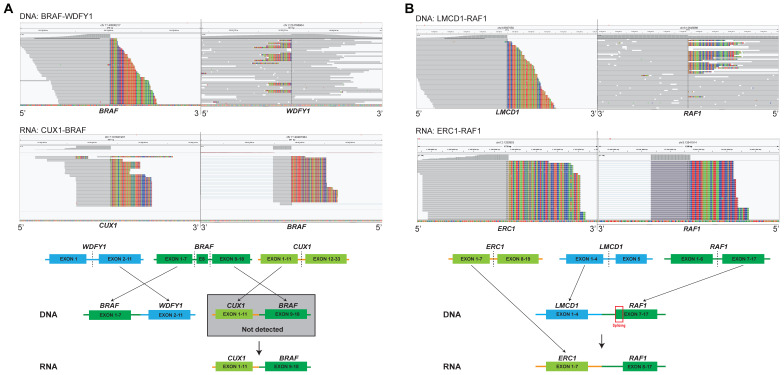
RNA sequencing can identify and correct the putative fusion transcripts of DNA sequencing. (**A**) *BRAF* fusion identified by both DNA-seq and RNA-seq; and (**B**) *RAF1* fusion identified by both DNA-seq and RNA-seq.

Two fusions were detected by both DNA-NGS and RNA-NGS; however, their partners were different (Figure 4), namely, *BRAF-WDFY1* to *CUX1-BRAF* and *LMCD1-RAF1* to *ERC1-RAF1* (Table S1, P1–P2). Patient 1 harbored a *BRAF-WDFY1* fusion detected by DNA NGS with “antisense rearrangements” juxtaposing the 5′ portion of the kinase genes and the 3′ portion of intergenic partners (Figure 4A). This indicated that *BRAF* lacked the tyrosine kinase domain (TKD), and the patient could not be referred to *RAF* tyrosine kinase domain (TKIs). However, the RNA-NGS results revealed that *CUX1* exon 11 was fused to *BRAF* exon 9 with an intact TKD. The inconsistency between the DNA and RNA results was perhaps due to the reciprocal rearrangement in which DNA-NGS detected only the opposite breakpoint downstream of *BRAF* rather than the primary junction constituting *CUX1* and *BRAF.* In Patient 2, DNA-NGS revealed that the partner *LMCD1* was fused to the 3′ portion of *RAF1* and that the breakpoint was in intron 9 of *RAF1*. At the RNA level, *CUX1-BRAF* was found, in which exon 8 of *RAF1* was involved (Figure 4B). Alternative splicing may contribute to functional expression.

### 3.5. Novel Fusions Detected in Melanoma Patients

**Figure 5:**
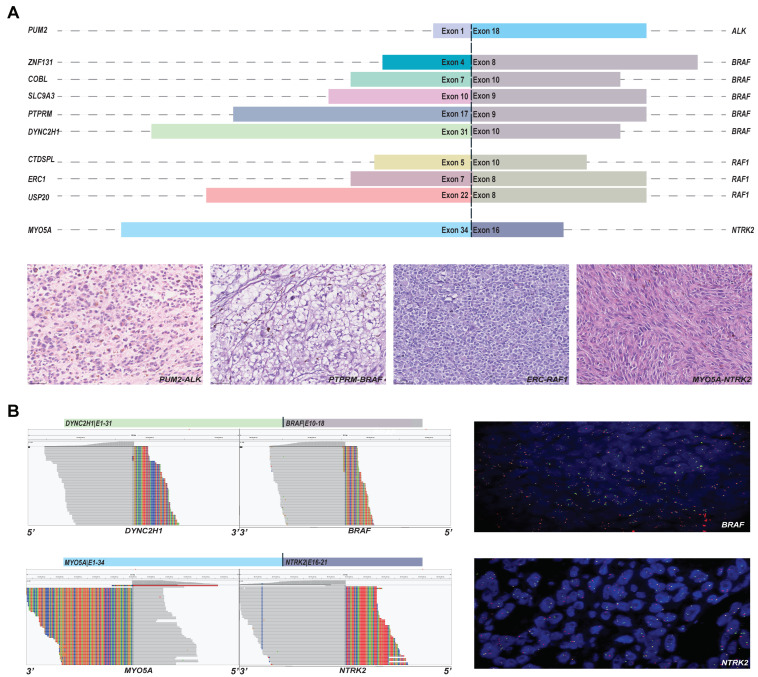
The novel fusions detected by panels. (**A**) The partners and drivers of novel fusions and representative H&E images; and (**B**) the further validation by FISH.

Ten novel potential druggable fusions were found in the cohort, which involved *ALK*, *BRAF*, *RAF1*, and *NTRK2* (Figure 5A). *SLC9A3-BRAF*, *PTPRM-BRAF*, and *DYNC2H1-BRAF* were identified by both DNA-NGS and RNA-NGS. *ERC1-RAF1*, *PUM2-ALK*, *ZNF131-BRAF*, *COBL-BRAF*, *CTDSPL-RAF1*, and *MYO5A-NTRK2* were identified by RNA-NGS only, although the breakpoint of *ERC1-RAF1* was covered by DNA-NGS. *USP20-RAF1* was identified and sequenced only by DNA-NGS due to the limited amount of specimen. The H&E staining images of each fusion are presented in Figure 5A and Figure S1. Samples with enough specimens were further validated by FISH, and break-apart signals were found in both *DYNC2H1-BRAF* and *MYO5A-NTRK2* (Figure 5B).

## 4. Discussion

In this study, the genetic landscape was systematically analyzed in a Chinese melanoma cohort. Seventy-five percent of Chinese melanoma patients were found to be targetable, and the predominant druggable gene was *BRAF* based on DNA-NGS. Furthermore, RNA-NGS identified actionable fusions in 11.67% of patients in the undruggable group, which further enhanced the number of druggable patients from 75% to 78%. These results demonstrated the importance of integrated DNA and RNA sequencing in improving druggable patients with melanoma, which could accurately guide personalized melanoma therapy.

Melanoma has been studied extensively in the Caucasian population [7,17,18,19,20], but only a few studies with limited cohort sizes could be found in Asia [3,21,22,23,24]. Annotated by the OncoKB database, the results from the MSKCC and AACR studies indicated that approximately 60% of Caucasian melanoma patients could be treatable after DNA-NGS testing [14,15]. In contrast, Park et al. discovered that DNA-seq could help approximately 40% of Asian melanoma patients [25]. It seems that there is a pathological split between Caucasian and Asian populations. However, around 75% of Asian melanoma patients could be treatable with medication based on our findings. TMB-H (20.5%), annotated as L1 in OncoKB, was analyzed in the cohort and may contribute to the high actionable proportion. Nonetheless, around 55% patients were druggable after excluding TMB-H patients. Furthermore, up to 5% of patients in the cohort had fusions with potential clinical significance, which may be another reason. To our knowledge, druggable fusions in Asian melanoma have not yet been characterized. No fusions were reported in the largest known Asian cohort (*n* = 178) from the Park et al. study [22]. Only one *BRAF* fusion and one *ALK* fusion were identified in the largest Chinese cohort (*n* = 81) from the Huang et al. study [21]. In contrast, 16 fusions with potential clinical significance were detected in our study, which is the largest Chinese melanoma cohort (*n* = 469). The leading number of fusions with clinical significance in our cohort was that of *BRAF* (1.7%, 8/469), which was slightly higher than that in the Caucasian population (1.1%) [14]. It is interesting to note that in contrast to glioma, *KIAA1549-BRAF* is the most common *BRAF* fusion [26]. The partners of *BRAF* fusions in our present study were discrete. This result demonstrated that there was no enriched *BRAF* fusion type in melanoma. Furthermore, the *RAF1* fusions (0.4%, 2/469) in the same pathway as *BRAF* were first reported in the Asian cohort. Among these fusions, four (three *BRAF* fusions and one *RAF1* fusion) were novel. Additionally, our study identified *NRG1* fusions in melanoma, which have never been found before. Surprisingly, we found high numbers of *ALK* rearrangements in melanoma (*n* = 4) for the first time. However, compared to the results in lung cancer [27], the partners of the *ALK* fusions in our study were totally different, and none of them were the canonical type (*EML4-ALK*). Couts et al. demonstrated that melanoma expressing *EML4-ALK* was sensitive to *ALK* inhibitors in vitro and in vivo [28]. This result emphasized the widespread beneficial potential for melanoma patients with *ALK* fusions from our study.

Although more than half of the population could be targetable based on DNA-NGS results from our findings and the abovementioned studies [14,15], approximately 25% to 42% of patients were still nondruggable. Benayed et al. found that extra fusions were identified by RNA-NGS in 14% of NSCLC patients from a driver-negative group, and 91.6% of the fusions were targetable [16]. This suggested that RNA-NGS is an effective way to expand the druggable targets by identifying actionable fusions. However, few studies have explored how RNA-NGS is essential in enhancing the druggable proportion in melanoma. Hutchinson et al. [7] reported only two fusions from a driver-negative melanoma cohort, which was limited to *BRAF* fusions. In our study, RNA-NGS sequencing was systemically performed on patients without potential clinical utility at the DNA level for the first time, and comprehensive fusion types were investigated. In addition to the *BRAF* fusions, *ALK*, *RAF1*, and *FGFR1* fusions were detected in the cohort. Briefly, up to 11.7% (7/60) of patients in the undruggable group could benefit from RNA-NGS based on the results. Overall, the use of RNA-NGS increased the proportion of treatable patients from 75% to 78%.

The increased proportion of fusion detection by RNA-seq could be attributed to several causes. Large introns could not be covered by the panel assay due to their length. For example, intron 13 of *NTRK3* is close to 100 kb in length (UCSC Genome Browser), which is close to 10% of the total size of the panel. Tiling such introns is not only technically challenging but also not practical in terms of the overall sequencing throughput. Additionally, introns with low frequency will not be tiled due to the cost factor. For example, *ALK* fusions have been frequently found in introns 18, 19, and 20 [29]. To our knowledge, the *ALK* breakpoint in intron 17 was first identified in our study, so it is unrealistic to be covered as a frequent intron in gene rearrangement.

On the other hand, eight fusions only detected by RNA-NGS were expected to be identified by DNA-NGS. Due to the complexity of the introns, the rearrangement that takes place in an intron may not be successfully detected by DNA-NGS. Five of those fusions involved BRAF on introns 7, 8, and 9. Similar to ROS1, the repeat sequence in those introns can be present at many other sites in the genome, and the inclusion of baits for these regions would simply result in unmappable reads [16,30]. Therefore, genomic breaks in these regions are usually missed. To enhance the fusion detection rate, the introns of specific partners could be included in the panel. For example, *CD74*, the most frequent partner in the ROS1 fusion, is usually covered by the DNA-seq panel. However, the partners in the *BRAF* fusions were too diverse to be covered.

### Limitations

Several limitations should be discussed. The clinicopathological parameters, such as clinical subtypes and disease stages were missed in the study. Those parameters could bring more a comprehensive understanding of Asian melanoma. Due to limitations in the amount of specimens, only samples with enough specimens were further sequenced by RNA-NGS. The resulting increased proportion of potential druggable patients in the undruggable group may be biased. However, more than half of the samples in the undruggable group had been analyzed by RNA-NGS, so the results did not induce significant bias. In addition, this study was a retrospective study, and patients with positive fusion detected by RNA-NGS were not able to receive targeted therapy, especially those with novel fusions with potential clinical significance. Therefore, further prospective trials of applying DNA and RNA sequential sequencing in targeted therapy for melanoma patients are needed in the future.

## 5. Conclusions

This study systemically analyzed the genetic landscape of Asian melanoma and demonstrated how DNA and RNA sequential sequencing is essential in bringing clinical benefits to more melanoma patients.

## Data Availability

All data used in this study were available in the main text and Supplementary Materials.

## Supplementary Materials

The following supporting information can be downloaded at: https://www.mdpi.com/article/10.3390/cancers15010283/s1, Figure S1. The representative H&E images of novel fusions. Table S1. The comparison of fusions at DNA and RNA levels in the melanoma patients.

## List of Abbreviations

NGS	Next-generation sequencing
TKI	Tyrosine kinase inhibitor
TMB	Tumor mutation burden
MSI	Microsatellite instability
NSCLC	Non-small-cell lung cancer
FFPE	Formalin-fixed paraffin-embedded
