# Supplementary material for: Genomic and Transcriptional Profiling of Chinese Melanoma Patients Enhanced Potentially Druggable Targets: A Multicenter Study
Source: Cancers (Basel). 2022 Dec 31;15(1):283. doi: 10.3390/cancers15010283 (PMC9818204; doi:10.3390/cancers15010283)
Supplement: Supplementary file 1 [file cancers-15-00283-s001.zip › cancers-2071900-supplementary.pdf]

Genomic and transcriptional profiling of Chinese melanoma patients enhanced potentially druggable targets: a multicenter study

Yue Li[1*], Baoming Wang[2*], Chunyang Wang[2], Dandan Zhao[2], Zhengchuang Liu[3], Yanling Niu[2], Xiaojuan Wang[2], Wei Li[2], Jianhua Zhu[2], Houquan Tao[3,4], Tonghui Ma[2,3#], Tao Li[5#]

[1] Harbin Medical University Cancer Hospital, Harbin, China

[2] Jichenjunchuang Clinical Laboratory, Hangzhou, Zhejiang, China.

[3] Key Laboratory of Gastroenterology of Zhejiang Province, Zhejiang Provincial People's Hospital, People's Hospital of Hangzhou Medical College, Hangzhou 310014, Zhejiang, China.

[4] Department of Surgery, Zhejiang Provincial People's Hospital, People's Hospital of Hangzhou Medical College, Hangzhou 310014, Zhejiang, China.

[5] Department of Bone and Soft-tissue Surgery, The Cancer Hospital of the University of Chinese Academy of Sciences(Zhejiang Cancer Hospital), Institute of Basic Medicine and Cancer(IBMC)，Chinese Academy of Sciences, Hangzhou, Zhejiang 310022, P.R. China

* Joint first authors
# Joint corresponding authors
Tonghui Ma　tonghuima@yeah.net
Tao Li　litao@zjcc.org.cn

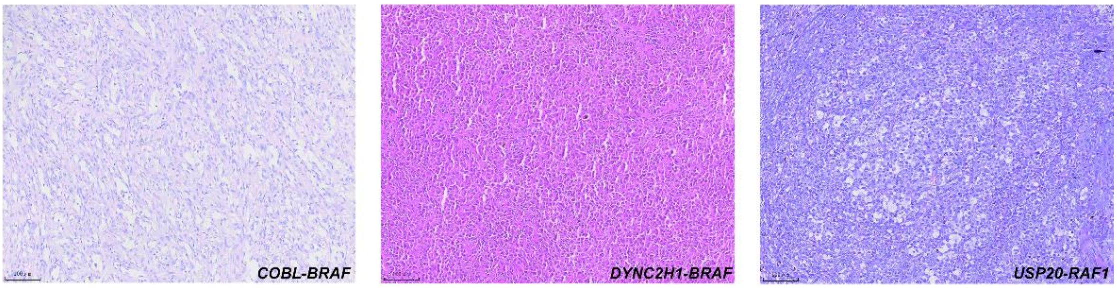

Figure S1. The representative H&E images of novel fusions.

Table S1. The comparison of fusions at DNA and RNA levels in the melanoma patients

ID	DNA NGS data			RNA NGS data		
	Gene1-Gene2	Exon1-Exon2	Frequency	Gene1-Gene2	Exon1-Exon2	Frequency
P1	BRAF-WDFY1	E7:E2	13.77	CUX1-BRAF	E11:E9	1.97
P2	LMCD1-RAF1	E4:E7	14.24	ERC1-RAF1	E7:E8	6.16
P3	DCTN1-ALK	E26:E20	10.01	DCTN1-ALK	E26:E20	93.3
P4	DYNC2H1-BRAF	E31:E10	18.2	DYNC2H1-BRAF	E31:E10	34.71

P5	TPM3-ALK	E7:E20	4.8	TPM3-ALK	E7:E20	1.51
P6	PTPRM-BRAF	E17:E9	4.8	PTPRM-BRAF	E17:E9	38.46
P7	SLC9A3-BRAF	E10:E9	0.8	SLC9A3-BRAF	E10:E9	50.98
P8	USP20-RAF1	E22:E8	6.4	N/A		
P9	DCTN1-ALK	E26:E20	13.4	N/A		
	ALK-NBAS	E19:E52	13.4	N/A		
P10	BRAF-NRF1	E8:E9	23.72	N/A		
P11	CHCHD3-BRAF	E7:E11	37.88	N/A		
	CADPS2-TERT	E3:E2	9.6	N/A		
P12	GTF2I-BRAF	E11:E10	8.8	N/A		
P13	NSMCE2-TERT	E5:upstream	7.1	N/A		
P14	TPPP-TERT	E2:E3	11.1	N/A		
P15	ADCY2-TERT	E20:upstream	13.6	N/A		
P16	RELA-DONSON	E4:E5	5.1	N/A		
	RELA-ENAH	E4:E2	5.1			
P17	AGAP3-BRAF	E10:E9	25.5	N/A		
P18	NRG1-SORL1	E5:E33	9.2	N/A		
P19	SLC2A13-NAB2	E1:E2	NA	N/A		
P20	NAB2-PDE1B	E5:E2	10.2	N/A		
P21	FZD3-NRG1	E2:E6	19.5	N/A		
	BCR-GSTT4	E8:E2	12			
P22	ADCY2-TERT	E22:upstream	16.7	N/A		
P23	CTNND2-TERT	E17:upstream	36.2	N/A		
P24	Negative			ZNF131-BRAF	E4:E8	10.69
P25	Negative			SOX6-RAF1	E9:E8	1.5
P26	Negative			COBL-BRAF	E7:E10	34.73
P27	Negative			AKAP9-BRAF	E22:E9	6.4
P28	Negative			ZKSCAN1-BRAF	E1:E10	43.02
P29	Negative			AGK-BRAF	E2:E8	4.34
P30	Negative			MYO5A-NTRK2	E34:E16	41.84
P31	Negative			FGFR1-TACC1	E17:E7	23.88
P32	Negative			CTDSPL-RAF1	E5:E10	11.21
P33	Negative			AGAP3-BRAF	E10:E3	22.28
P34	Negative			PUM2-ALK	E1:E18	28.21

P35	Negative	MYO5A-NTRK3	E33:E14	31.14
P36	Negative	SMARCB1-BCR	E7:E2	2.82
P37	Negative	PAX5-BAG1	E4:E2	20.21
P38	Negative	TCF12-GLDC	E8:E6	4.87
P39	Negative	FNDC3A-RB1	E7:E18	16.01
P40	Negative	GRIP1-ATF1	E3:E14	2.91
P41	Negative	ETV6-CDH18	E1:E3	1.47
P42	Negative	MAG-CD22	E10:E1	9.02
P43	Negative	SNAP29-MRTFA	E5:E3	1.6
P44	Negative	ETV6-RP11-513 G19.1	E2:E4	1.31
P45	Negative	WHSC1L1-TRP M1	E19:E3	2.58
P46	Negative	JAZF1-SEPTIN7	E1:E8	4.73
P47	Negative	CBFB-CES4A	E3:E2	3.82
P48	Negative	KDM6A-VAPB	E18:E2	4.56
P49	Negative	ETS1-ARHGEF2	E1:E2	7.57
P50	Negative	ETS1-STT3A	E1:E3	1.08
P51	Negative	ZCCHC7-MELK	E5:E2	1.69
P52	Negative	JAZF1-AC00489 5.4	E1:E3	3.5
P53	Negative	AKT3-NAV1	E2:E5	1.13
P54	Negative	NF1-MED1	E36:E17	20.61

NA: Not detected
